# Supplementary material for: Factors Associated with Variability in Pulse Wave Transit Time Using Pulse Oximetry: A Retrospective Study
Source: J Clin Med. 2022 Jul 7;11(14):3963. doi: 10.3390/jcm11143963 (PMC9319785; doi:10.3390/jcm11143963)
Supplement: Supplementary file 1 [file jcm-11-03963-s001.zip › jcm-1774329-supplementary.pdf]

Journal of Clinical Medicine

MDPI

The relationship between ΔPWTT and ΔSBP was determined using Spearman's correlation. This analysis showed a statistically significant positive correlation between PWTT variability and SBP change ($r_s = 0.196$, $p < 0.001$) (Supplementary Table S1).

Table S1. Spearman's correlation analysis of △PWTT and △SBP.

Variables		△PWTT	△SBP	95% Confidence Intervals (2-tailed) [a,b]
△PWTT	**Correlation Coeff. (r_s)**	**1.000**	**0.196 ****	(0.106–0.283)
	p value (2-tailed)		0.000	
	N	485	480	

** Correlation is significant at the 0.01 level (2-tailed). [a] Estimation is based on Fisher's r-to-z transformation. [b] Estimation of standard error is based on the formula proposed by Fieller, Hartley, and Pearson.

Multiple regression model to validate the association of ΔPWTT with clinical characteristics, using the same data to confirm the factors influencing ΔPWTT obtained from the logistic regression analysis. Age, LA cartridge, respiratory disease, and phobia were statistically significant factors for PWTT variability (Supplementary Table S2).

Table S2. Multiple regression of ΔPWTT with clinical characteristics.

Variables	*B* (sd)	*p* Value
(Constant)	128.709 (64.336)	2.001
Age (years)	-0.155 (0.049)	-3.186
L.A cartridge (1.8mL)	6.800 (0.74)	9.187
Other Respiratory Ilness (y/n)	7.949 (3.547)	2.241
Phobia (y/n)	-3.221 (1.455)	-2.214
